# Ultra-Long-Term-EEG Monitoring (ULTEEM) Systems: Towards User-Friendly Out-of-Hospital Recordings of Electrical Brain Signals in Epilepsy

**DOI:** 10.3390/s24061867

**Published:** 2024-03-14

**Authors:** Gürkan Yilmaz, Andrea Seiler, Olivier Chételat, Kaspar A. Schindler

**Affiliations:** 1 Swiss Center for Electronics and Microtechnology (CSEM SA), 2002 Neuchâtel, Switzerland; 2 Sleep-Wake-Epilepsy Center, Department of Neurology, Inselspital Bern, University Hospital, University of Bern, 3010 Bern, Switzerland

**Keywords:** wearables, electroencephalography, epilepsy, sleep, dementia

## Abstract

Epilepsy is characterized by the occurrence of epileptic events, ranging from brief bursts of interictal epileptiform brain activity to their most dramatic manifestation as clinically overt bilateral tonic–clonic seizures. Epileptic events are often modulated in a patient-specific way, for example by sleep. But they also reveal temporal patterns not only on ultra- and circadian, but also on multidien scales. Thus, to accurately track the dynamics of epilepsy and to thereby enable and improve personalized diagnostics and therapies, user-friendly systems for long-term out-of-hospital recordings of electrical brain signals are needed. Here, we present two wearable devices, namely ULTEEM and ULTEEMNite, to address this unmet need. We demonstrate how the usability concerns of the patients and the signal quality requirements of the clinicians have been incorporated in the design. Upon testbench verification of the devices, ULTEEM was successfully benchmarked against a reference EEG device in a pilot clinical study. ULTEEMNite was shown to record typical macro- and micro-sleep EEG characteristics in a proof-of-concept study. We conclude by discussing how these devices can be further improved and become particularly useful for a better understanding of the relationships between sleep, epilepsy, and neurodegeneration.

## 1. Introduction

As recent studies have impressively demonstrated, epilepsy is a dynamical disease with patient-specific temporal patterns of pathological brain activity on many different, i.e., ultra-, circa- and multidien scales [1,2]. However, the current standard practice of monitoring patients in an appointment-based way with snapshot-like assessments of seizure reports, clinical signs, symptoms, and electroencephalographic recordings (EEG) does not allow for accurate tracking of the dynamics of epilepsy [3]. To close this crucial information gap, user-friendly devices that allow for the recording of electrical brain signals under real-world conditions in patients’ everyday out-of-hospital lives offer promising prospects [4]. Importantly, these ambulatory EEG devices have to be as unobtrusive and thus non-stigmatizing as possible, as sadly stigmatization is still a severe challenge for people with epilepsy [5]. Though implantable EEG devices are hardly or not-at-all visible once surgery is accomplished [6,7], due to their invasiveness they are highly likely to remain restricted to patients suffering from pharmaco-resistant epilepsies for the foreseeable future. Therefore, it is important to design EEG systems that are both non-invasive and less stigmatizing [8,9,10]. Stigmatization may be reduced by integrating the device into objects of daily use or restricting recordings to nighttime sleep when patients are not socially exposed. Here, we describe two versions of an ultra-long-term EEG monitoring system, referred to as ULTEEM and ULTEEMNite, respectively, that follow these approaches to decrease stigmatization. ULTEEM is a single-lead EEG acquisition system consisting of two sensors, which can simply be clipped onto a patients’ eyeglasses to record electrical brain signals from the temporal brain regions. This clip-on solution empowers patients to remove the sensors whenever they feel uneasy [11]. To connect the two sensors electrically, ULTEEM makes it possible to use the metal frames of the glasses as they are, i.e., not necessarily insulated or shielded. However, thanks to a special unconventional circuit, there is no degradation of the signal quality. Furthermore, its gel-free, dry electrodes increase usability without compromising on signal quality or reliability. ULTEEMNite, the complementary version of ULTEEM, allows for recording electrical brain signals from frontal brain regions during sleep. ULTEEMNite is positioned on the forehead by an adjustable headband and is equipped with dry electrodes, too. 

One aspect hindering the widespread use of wearable devices for medical applications is that most of the devices in the market have been developed for consumer applications. Therefore, they do not meet the performance requirements for medical devices, for example, in regard to signal quality and reliability. Consequently, physicians do not—and are not allowed to—rely on signals acquired by such devices for diagnostics or the monitoring of therapies. To address this shortcoming, both the ULTEEM and ULTEEMNite devices were designed in accordance with the medical device standards regulating clinical EEG systems. In addition, ULTEEM and ULTEEMNite were verified to satisfy the requirements of electrical safety as defined in IEC 60601-1 [12]. Upon successful performance and electrical safety verification of the ULTEEM device, a pilot study was carried out with 10 healthy volunteers for clinical assessment. Electrical brain signals were recorded simultaneously with ULTEEM and with a certified EEG device during different states. Visual analysis by expert neurologists followed by statistical analysis showed good accuracy. Moreover, we here present a whole-night-sleep recording from a healthy subject using the ULTEEMNite device. We demonstrate that its signal quality easily allows one to identify typical sleep macro- and micro- EEG patterns such as sleep cycles, spindles, and slow waves.

We conclude by discussing potential future improvements and clinical applications of the ULTEEM and ULTEEMNite systems.

## 2. Materials and Methods

**Figure 1:**
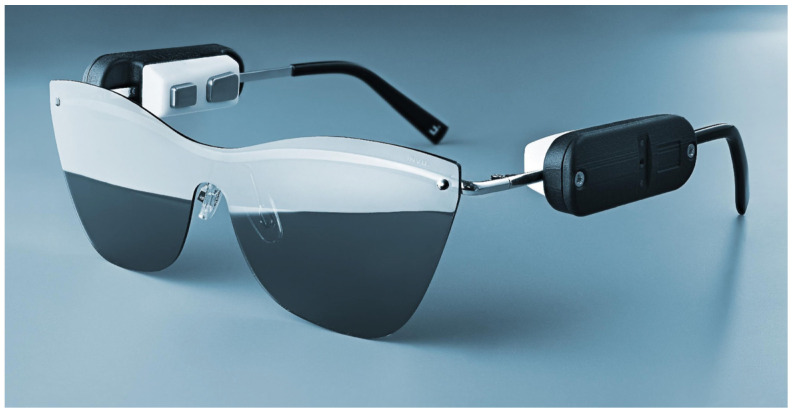
ULTEEM device consisting of sensors with active dry electrodes clipped onto the metallic frame of the eyeglasses.

**Figure 2:**
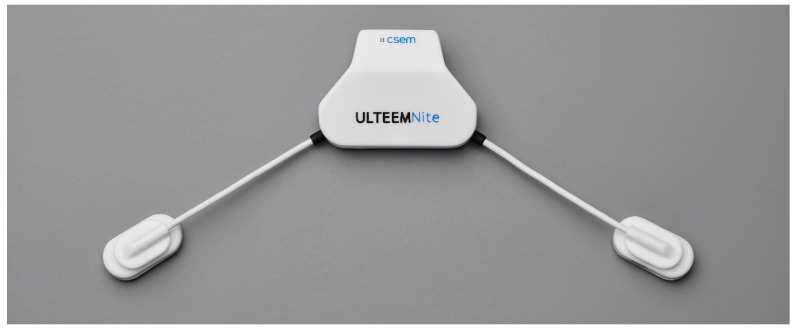
ULTEEMNite device with its central unit where processing, storage, and power management units are located, and the two sensor nodes equipped with active-dry electrodes.

As outlined above, there is an urgent need for user-friendly wearable devices that allow the recording of electrical brain signals under real-world conditions for prolonged periods and that meet the requirements for medical certification. Though medical grade ambulatory EEG devices exist and are used for up to a week duration, they remain bulky and uncomfortable to wear, with many cables and the need to apply gel or glue to the electrodes. On the other hand, wearable devices or gadgets targeting consumer applications perform better in terms of usability, yet they fall short in addressing the signal quality and reliability needed for medical applications. Therefore, we set out to design medical-grade devices with improved usability. We developed two different yet complementary devices to monitor electrical brain signals during day and night: (i) the ULTEEM system that can be clipped on the metallic frame of eyeglasses (Figure 1) and (ii) the ULTEEMNite system that can be worn integrated in a headband during sleep (Figure 2). This section details the design of these two single-lead EEG devices aimed to measure brain signals in an out-of-hospital setting for prolonged time periods.

Both devices were developed according to the requirements (Table 1) derived from the relevant medical electrical equipment standards. For general safety and essential performance, IEC 60601-1 [12] (with its latest amendments) was considered. More precisely, patient auxiliary current requirements for Type BF (Table 1) were derived from [12] to ensure the basic safety of the devices when used on humans. EEG-related performance requirements (bandwidth and input-referred noise within this bandwidth) were derived from IEC 60601-2-26 (particular requirements for the basic safety and essential performance of electroencephalographs) [13] (Table 1): the standard requires a bandwidth at least between 0.5 and 50 Hz and that the input-referred noise shall not exceed 6 µV peak-to-peak. Since both devices use dry and not gel electrodes, two important requirements have been defined based on our previous experiences [14] and the literature [15,16], namely, (i) input impedance and (ii) input leakage current. While the EEG standard [13] does not specify an input impedance requirement, the input impedance specification recommended in IEC 60601-2–25 [17], the most stringent electrocardiogram (ECG) standard, remains very low for dry electrodes. It is worth noting that these standards consider skin–electrode impedance values which rather reflect the utilization of gel electrodes. Specifically, ref. [17] indicates that the input impedance (Z_in_) has to be higher than 2.5 MΩ at 10 Hz. However, our experience [14] and the literature [18] indicate that the input impedance of Z_in_ should be significantly higher than this value if the EEG front-end circuit is to be interfaced to the skin via dry electrodes, more precisely at least 0.5 GΩ at 10 Hz. Input leakage current (I_b_) is not specifically indicated, neither in the EEG standard [15] nor in the ECG standard [17], as this parameter is less of a concern with gel electrodes—except that the general standard [12] specifies patient auxiliary currents for direct current (DC) and alternating current (AC). However, again due to the high interface impedance introduced by dry electrodes, this parameter becomes an important part of the design. Consequently, input leakage current (I_b_) was specified to be smaller than 100 pA, in the same order of magnitude as in the literature [18] in which the authors aim to specify input leakage current for designs incorporating dry electrodes. Table 1 summarizes the most important requirements for the electronics design of the ULTEEM and ULTEEMNite devices.

**Figure 3:**
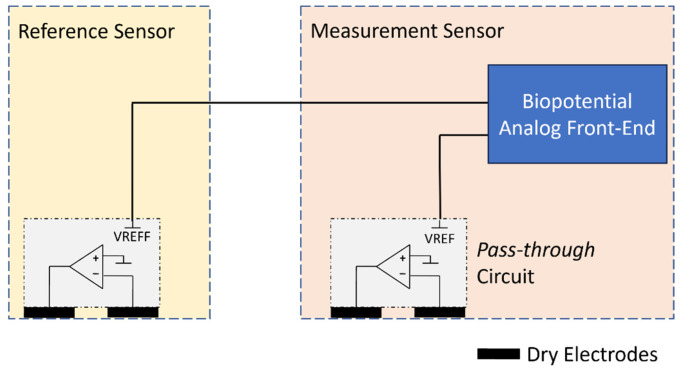
Simplified block diagram for biopotential measurement using a single wire between a measurement sensor and a reference sensor using a pass-through circuit. The pass-through circuit is implemented by an operational amplifier.

The ULTEEM and ULTEEMNite devices share the same high-level electronics design. Therefore, in the following, only ULTEEM is presented in detail. The ULTEEM device is composed of two sensors, a measurement sensor and a reference sensor. The reference sensor is equipped with a so-called *pass-through* circuit [19] and its two stainless-steel electrodes as well as power management circuitry, i.e., battery charger, a low drop-out voltage regulator, and a rechargeable battery. The pass-through circuit creates a virtually zero-impedance path between the wire connecting the two sensors and the body part, which is located just underneath the high-impedance voltage-measurement electrode of the reference sensor (see dry electrode connected to the inverting input of the operational amplifier in Figure 3). Importantly, the *pass-through* circuit allows for connection of the two sensors with an unshielded and uninsulated wire with no degradation of performance [19], which is particularly crucial for the operation of ULTEEM on an arbitrary metallic frame. The operation principle of the *pass-through* circuit is detailed in a prior publication by our group [19], and its electrocardiogram performance is reported in [20]. The main differences between an ECG acquisition system and an EEG acquisition system are (i) the acquisition bandwidth, (ii) compatibility with different amplitude ranges, and (iii) the noise performance in the acquisition bandwidth. The *pass-through* circuit practically does not impact the first two parameters. An operational amplifier’s bandwidth is much wider than the acquisition bandwidth of ECG and EEG systems. An EEG system requires an input dynamic range of ±0.5 mV, while an ECG system requires an input dynamic range of ±5 mV. Therefore, EEG systems can benefit from higher gain at secondary stages. The third parameter, input-referred noise, is the most critical parameter to make sure that the *pass-through* circuit is compatible with EEG recordings. Accordingly, a low-noise operational amplifier was selected (see Table 2 for verification results).

The measurement sensor is a superset of the reference sensor with the addition of an analog front-end (AFE) module and digital circuits. The AFE module performs signal acquisition and conditioning as well as analog-to-digital conversion. Digital circuits are responsible for on-board processing, storage, wireless connectivity, and power management. In Figure 3, a simplified block diagram is displayed showing the biopotential measurement and the implementation of the *pass-through* circuit by an operational amplifier (opamp). The selection of the operational amplifier is an important step towards satisfying the design requirements, particularly for input leakage current and input-referred noise. The *pass-through* circuit also implements a power supply bootstrap, resulting in an electrode input impedance equal to the opamp input impedance multiplied by the opamp gain [19], which is of the order of 100,000 at the EEG frequencies. We used an LTC6078 (Analog Devices, Wilmington, MA, USA) for the pass-through operational amplifier. In order to reduce the footprint of the signal conditioning and quantization on the printed circuit board (PCB) surface, we opted for a complete analog front-end solution with integrated ADC: MAX30001 (Analog Devices, Wilmington, MA, USA). However, the front-end performance of this chip in terms of leakage current and input impedance is irrelevant, as these are determined solely by the *pass-through* circuits.

Both the ULTEEM and ULTEEMNite devices are equipped with a system-on-chip (SoC), which functions as the application processor and supports the Bluetooth Low Energy (BLE) protocol stack (nRF52840 from Nordic Semiconductor, Norway). Acquired signals are stored on an on-board non-volatile memory (1 Gb NOR Flash from Micron Technology, Boise, ID, USA) and can be streamed to a portable device (e.g., a smartphone, tablet, or PC) via BLE. An application interface running on Android OS was developed to visualize the recorded signals in almost-real time and provide the users with feedback, such as remaining battery percentage, available memory space, and connectivity status. The stored data can then be downloaded by two means: (i) via BLE either already during recording or then after the recording session is completed and (ii) via USB communication after the recording session is completed. The latter option has a higher download speed and brings the additional usability benefit of simultaneously recharging the battery. For safety reasons, the headband does not allow the USB cable to be mechanically connected to the device when inserted into the headband. This measure implements the MOPP (measure of patient protection) required by IEC 60601-1 [12] for BF-type medical devices.

While the electronic designs of the ULTEEM and the ULTEEMNite systems are highly similar, their mechanical designs are significantly different. Both devices were designed by focusing on the goal of long-term monitoring. To achieve this, patient acceptance and adherence are essential [21]. However, patient expectations from devices worn during the daytime or nighttime are different. During the daytime, it is important that the device is ideally unnoticeable or at least easily removable. On the other hand, for nighttime use, this concern is replaced with having a comfortable and light-weight device, which does not interfere with sleep.

The ULTEEM device addresses this need thanks to its clip-on solution: the two sensors are not an integral part of the eyeglasses. This is a key difference of ULTEEM compared to devices generally referred to as smart glasses, in which sensors are integrated to the eyeglasses. The design of ULTEEM allows the patients to remove the sensors when they feel uncomfortable. This feature is primarily possible due to the measurement technology [22], which allows for connecting two sensors with an unshielded and uninsulated wire. The technology therefore allows use of the metallic frames of the eyeglasses to electrically connect the two sensors—which is necessary to measure a potential difference between two points.

**Figure 4:**
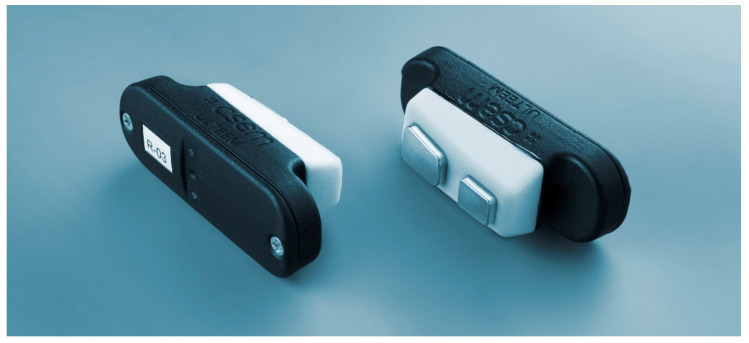
A close-up of a pair of ULTEEM clip-on sensors. The white part containing the stainless-steel dry electrodes and a spring backing is formed with a tapering to better adjust to the anatomy of an individual’s temple.

Dry electrodes are known [15] to be more susceptible to movement artefacts, and a stable contact between the dry electrodes and the skin is mandatory to achieve high-quality signal acquisition. An important anatomical challenge in our use case is that the morphology of the temple is slightly different for each individual. The ULTEEM design addresses this by connecting the dry electrodes to a spring structure, which provides slight pressure to ensure stable contact between the electrodes and the skin for different anatomies. Furthermore, the electrodes are formed with a tapering, which allows them to conform well to the temple (see the white part with varying thickness in Figure 4). The varying distance between the frame and the skin along the frame axis is therefore compensated for by design. The tapering also limits the movement range of the spring, directly constraining the final dimensions of the device.

The mechanical design of ULTEEMNite aims at achieving a light-weight device which is comfortable to sleep with. The first point to address was the placement of the device. Most people sleep either on their back or on their side, or they take turns [23]. Consequently, the central unit of the device, which contains all the electronics except the active dry electrodes, are placed at the center of the forehead. Contrary to ULTEEM, the electrodes are placed in more frontal positions to avoid discomfort for patients lying on their sides. Flexible cables connecting the central unit to the sensors (i.e., the active dry electrodes) enable placing of the sensors freely. Therefore, patients can make alignments to improve their comfort.

The ULTEEMNite device is held in place and applied on the forehead skin by a headband. The central unit is placed into a pocket and the sensors are fixed to the headband by inserting them into pre-cut slits. The headband is equipped with silicone anti-slip stripes to minimize sliding between the skin and the electrodes when the patient moves. The headband is fixed on the back of the head with a Velcro band. The headband is manufactured in two different sizes, with an overlap between the two sizes due to the length of the Velcro band.

The dry electrodes of ULTEEMNite (316L stainless steel) are manufactured by machining and over-molding with a medical grade acrylonitrile butadiene styrene (ABS). The cables linking the central unit and the sensors have biocompatible sheaths made from medical polyvinylchloride (PVC). Thus, all the parts in contact with the skin are fabricated from biocompatible materials. The central unit, which remains in the pocket and is not in contact with the body, is made from ABS-like polyurethane.

## 3. Results

In the following, the results obtained with the ULTEEM and ULTEEMNite devices are presented in three subsections: (i) verification of the devices, (ii) ULTEEM pilot clinical study, and (iii) proof-of-concept ULTEEMNite night-long recording.

### 3.1. Verification of the Devices

Following the manufacture and integration of the devices, the complete systems (i.e., with their housings in place) were tested to verify whether the design requirements (see Table 1) are satisfied. This subsection provides details about the verification methodology and results.

**Figure 5:**
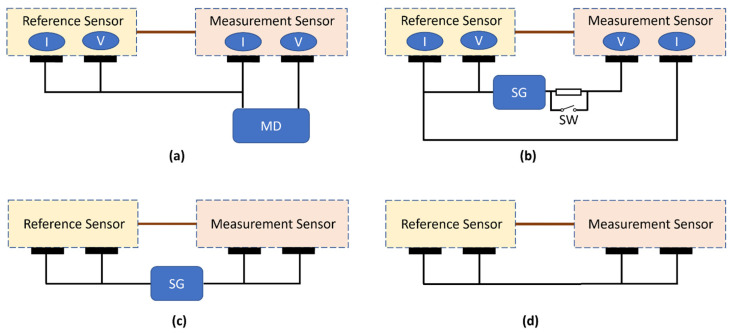
Test setup for verifications: (**a**) patient auxiliary current and input leakage current, (**b**) input impedance, (**c**) acquisition bandwidth, and (**d**) input-referred noise measurements. MD: measurement device, SG: signal generator, and SW: switch. I: low-impedance current-injection electrode, V: high-impedance voltage-measurement electrode.

**Patient auxiliary current:** Both devices are equipped with a total of four electrodes. Each sensor unit has a potential electrode (high-impedance input) and a current electrode (low-impedance input) so that currents, such as 50/60 Hz currents, can flow freely from the device to the body without passing through the potential electrode. Current must not flow through the potential electrode because the high electrode/skin impedance would convert this current into noise in the EEG signal. Patient auxiliary current (DC and AC) was measured for all combinations and both polarities. The measurement device (MD) was connected between one of the four electrodes and the three remaining electrodes, which were connected to each other (see Figure 5a). The measurements were performed with an electrical safety analyzer (Secutest SIII+, Gossen Metrawatt, Nürnberg, Germany) using the so-called Class III configuration, which refers to internally powered medical equipment. The type of the applied parts was selected as Type BF, according to the definition in [12]. The device self-reports the results and all the tests were successfully completed with a value <0.1 µA, which defines the minimum measurement limit of the device. As indicated above, to achieve high-quality signal acquisition with dry electrodes, the input leakage current (or patient auxiliary current for DC) needs to be much smaller than what the medical device standard defines. The more precise readings of the patient auxiliary current for DC can be found under input leakage current verifications.

**Lower and higher cut-off frequencies:** Within each sensor, the voltage-measurement electrode and current-injection electrode were short-circuited (see Figure 5c). A sinusoidal waveform (200 µV peak-to-peak) was applied between these two nodes first at 5 Hz and then at 0.5 Hz and 50 Hz. The EEG standard [13] requires that the measured signals at 0.5 Hz and 50 Hz be within 71% to 110% of the signal obtained at 5 Hz. The measured output signal at 5 Hz was >190 µV peak-to-peak and >150 µV peak-to-peak at 0.5 Hz and 50 Hz, thus meeting the requirements.

**Figure 6:**
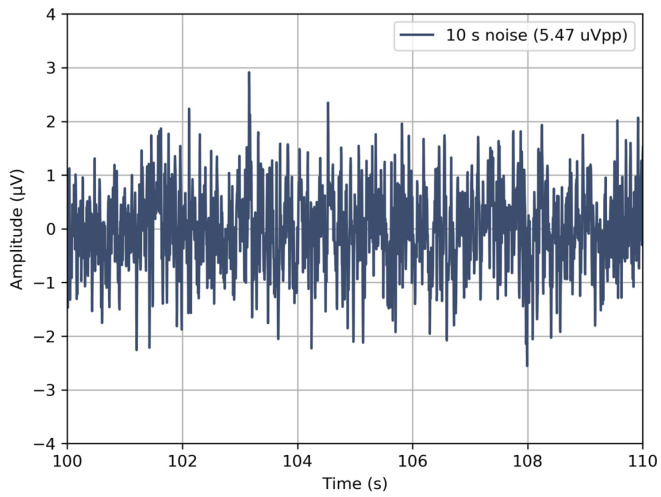
Input-referred noise of the devices within 0.5 and 50 Hz bandwidth for a 10-s recording. Only the worst result of 10 consecutive measurements is shown.

**Input-referred noise within acquisition bandwidth:** All the four electrodes were short-circuited, and ten measurements (see Figure 5d), each lasting 10-s, were performed. The acquired signals were filtered using a first-order Butterworth filter with a cut-off frequency of 0.5 Hz and 50 Hz, as described in the EEG medical device standard [13], and peak-to-peak values were recorded. The input-referred noise was calculated considering the gain and ADC conversion factors, and it was found to be less than 5.5 µV peak-to-peak (Figure 6), again satisfying the requirement.

**Input impedance at 10 Hz:** The ECG standard recommends a test circuit and method to measure input impedance (Z_in_): a series load is inserted in parallel with a switch between the signal generator and the device-under-test to create a voltage divider (see Figure 5b). The recorded output voltage is measured once with a series load (switch off position) and once without (switch on position). By this procedure, the input impedance of the system can be calculated. The ECG standard [17] defines the value of this series load as 620 kΩ in parallel with 4.7 nF, as the Z_in_ requirement is 2.5 MΩ. We modified the value of the series load to 6.2 MΩ in parallel with 0.47 nF, so that a measurable difference could be created for the target Z_in_ (0.5 GΩ) in our case. The measurements were performed for both sensors and on multiple devices. The input impedance was then calculated and found to range between 0.53 and 1.04 GΩ at 10 Hz, satisfying the requirement.

**Input leakage current:** Input leakage current was measured between the voltage-measurement electrode of one sensor and all the other three electrodes short-circuited (see Figure 5a). A digital multimeter (34470A, Keysight, Santa Rosa, CA, USA) was inserted between these two nodes, and a DC measurement was performed for both sensors. The measured values ranged between 39 and 71 pA for different PCBs and sensors.

Table 2 summarizes the results of the above verifications.

### 3.2. ULTEEM Pilot Clinical Study

An observational study was designed and conducted to evaluate the similarity between the EEG signals recorded by the ULTEEM device and by a medical EEG device. Specifically, EEG signals were simultaneously acquired by a reference EEG device (Natus Sleepworks, Brooklyn, NY, USA) and the ULTEEM device from 10 healthy subjects for 20 min. The reference EEG device used gel electrodes. The duration of the recording was defined based on the nominal and typical EEG recording duration in ambulatory clinical settings. Subjects were instructed to perform the following four actions during the recordings:

- keeping their eyes open and fixing their gaze as much as possible;
- keeping the eyes closed and relaxing;
- performing eye movements (left and right) without turning their head;
- swallowing repetitively.

The first three actions aimed at acquiring EEG signals with different profiles. The last action, on the other hand, was used to assess the impact of muscle and movement artefacts on signal acquisition.

Before each recording, the subjects were informed regarding the recording procedure via the study information, and their questions were answered. Upon reading, approving, and signing the informed consent form, the subjects were invited to the NeuroTec EEG labs to perform the recordings. The study was performed upon approval of the Kantonale Ethikkommission (KEK) Bern (see Institutional Review Board Statement).

**Figure 7:**
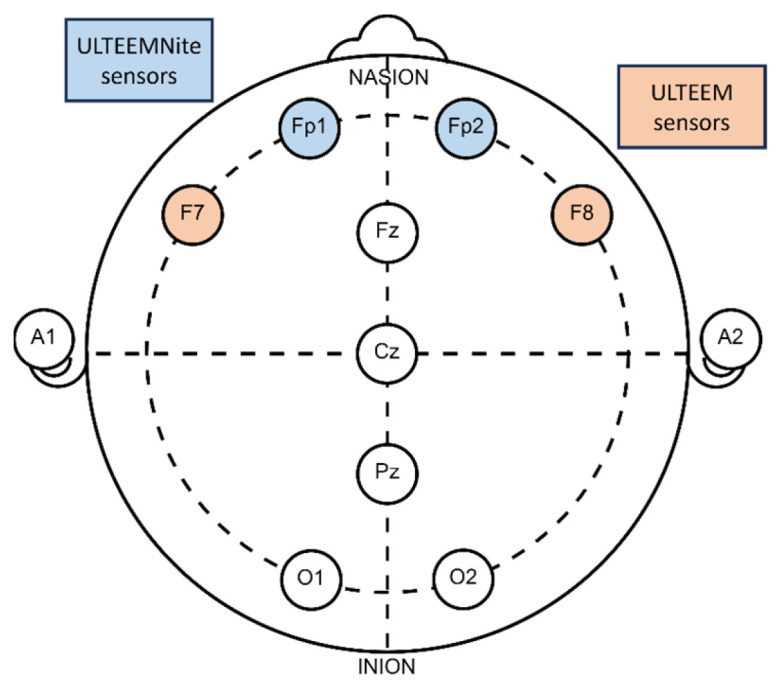
Placement of ULTEEM and ULTEEMNite devices with respect to the international 10–20 system. Position of ULTEEM device is marked with orange and of ULTEEMNite device with blue.

**Figure 8:**
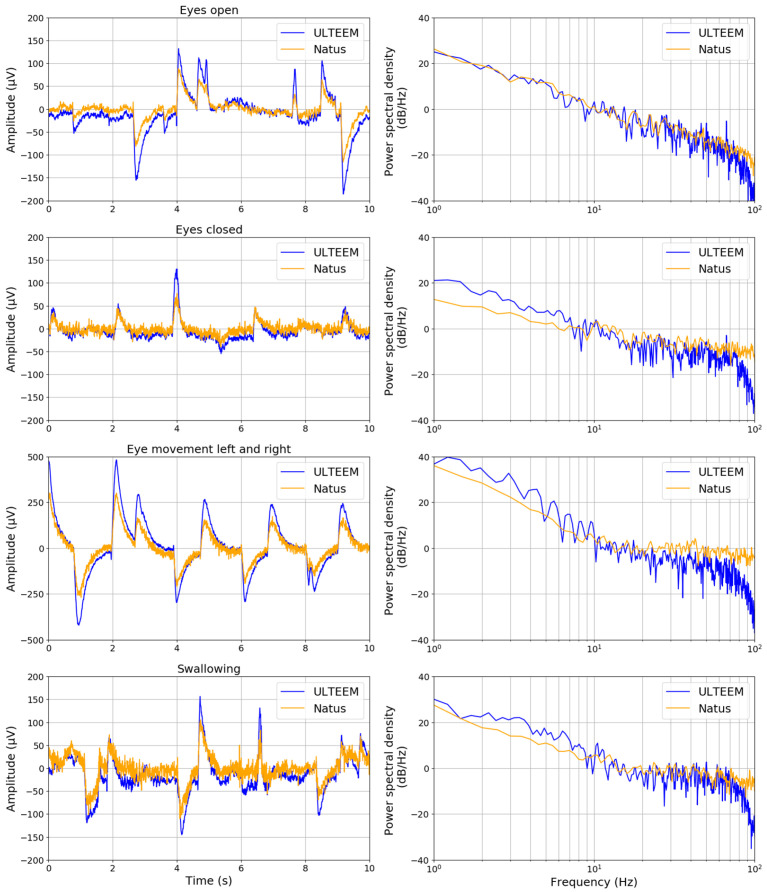
(**Left** column) Comparison of time-domain signals acquired under four different settings (eyes open, eyes closed, eyes moving to the left and right, and swallowing action while eyes are open) by the ULTEEM device (blue curve) and the reference EEG device (Natus SleepWorks) which uses gel electrodes (orange curve). (**Right** column) Comparison of frequency spectra of the corresponding time-domain signals for each action.

The ULTEEM device was placed at the so-called F7 and F8 locations, as defined by international 10–20 system (Figure 7). Figure 8 presents the time- and frequency-domain comparisons of the signals recorded by the ULTEEM device and the reference EEG system for the four different action tasks as described above for one of the subjects.

### 3.3. Proof-of-Concept ULTEEMNite Night-Long Recording

**Figure 9:**
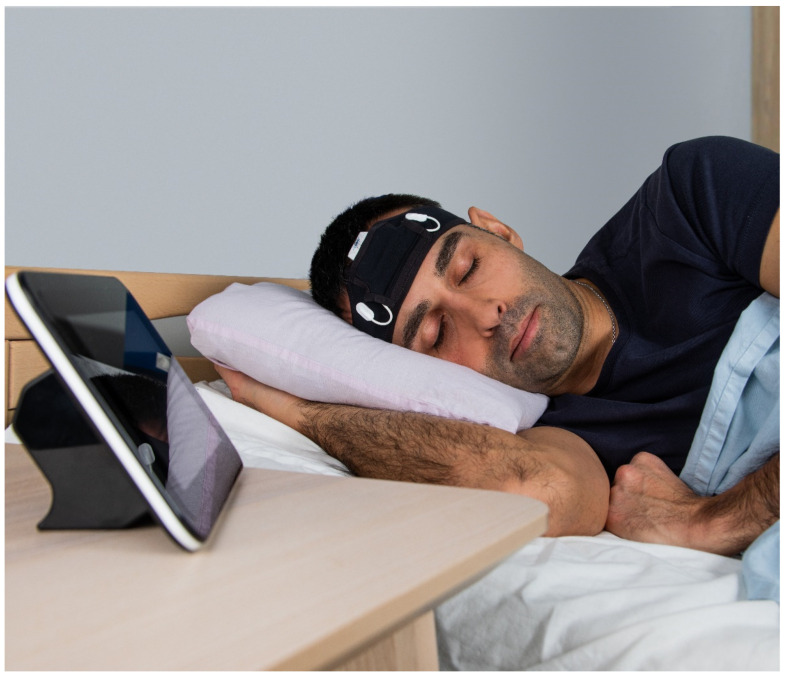
The ULTEEMNite system inserted into a headband and placed on the forehead. The central unit is slightly pulled out of its original place in the headbands pocket to increase its visibility. The device on the nightstand serves as a gateway to download recorded data from the ULTEEMNite device and to then upload it to a secure cloud server.

**Figure 10:**
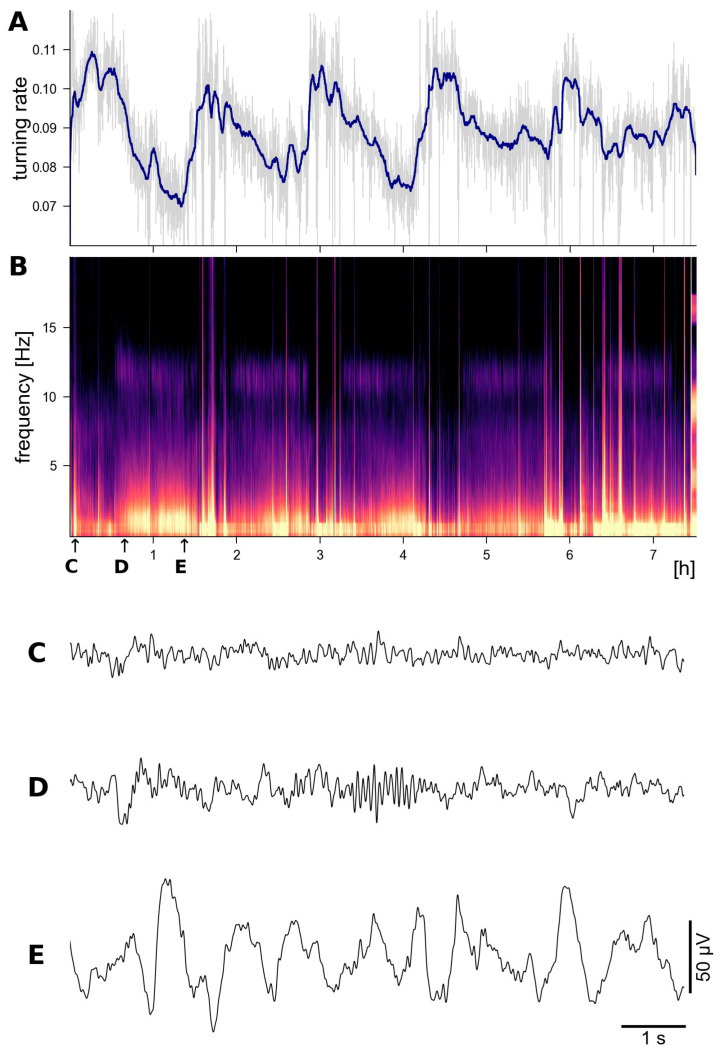
Night-long recording with ULTEEMNite in a healthy 53-year-old proband clearly reveals the typical macro-and microstructure of sleep EEG. In (**A**), the recorded EEG signal is analysed by computing the turning rate, which is defined as the probability of all non-monotonous ordinal patterns of length 3 [24,25] for moving windows of 10 s duration (in light gray). The blue line represents results from applying a 5 min moving median filter. The turning rate was proposed as a computationally efficient and robust time-domain-based measure to assess sleep depth, i.e., the lower the turning rate, the deeper the sleep. (**B**) shows a multi-taper spectrogram [26,27], revealing the typical spectral dynamics and motifs of five sleep cycles, i.e., periodically recurring power increases in the 0.5–4 Hz and 10–14 Hz frequency ranges. Representative EEG time segments of 10 s duration are shown for the non-REM sleep stage N1 in (**C**), stage N2 in (**D**) with typical sleep spindles, and for stage N3 with larger amplitude and more regular delta activity in (**E**). The times when these EEG segments were recorded are indicated by the corresponding labels and small arrows in (**B**).

The ULTEEMNite device was tested by a healthy proband for a night-long recording to assess its capability to detect typical macro- and micro-sleep EEG characteristics. Figure 9 shows a representative home recording setup, with the ULTEEMNite system inserted in a headband and placed onto the forehead. In this configuration, the ULTEEMNite device acquires a signal between the so-called Fp1 and Fp2 locations, as defined by the international 10–20 system (Figure 7). In Figure 10, a time-domain and a multi-taper spectral domain method were used to clearly visualize the dynamics of the five recorded sleep cycles. Furthermore, EEG signals from different non-rapid eye movement (non-REM) sleep stages are displayed, illustrating the high quality of the recording.

## 4. Discussion

The epileptic events that characterize epilepsy reveal patient-specific patterns on ultra- to multidien time scales [2,28]. To individualize and thus improve the diagnostics, monitoring, and therapy of epilepsy patients, we need user-friendly EEG devices that enable out-of-hospital recordings of electrical brain activity for long time periods. Therefore, we have developed the ULTEEM and ULTEEMNite devices. Here, we have presented their technical characteristics and demonstrated first, promising recordings during wakefulness and sleep.

As indicated in the usability factors impacting the ULTEEM design, the size of the sensors play an important role in increasing patient acceptance and adherence, particularly in the case of epilepsy patients. The current dimensions of the ULTEEM device still remain problematic for wearing in the public domain, and there is clearly room for improvement. Integrating the entire, or partial if it is more practical and cost-effective, electronics design in the form of an application-specific integrated circuit (ASIC) can significantly help reduce its dimensions. Moreover, moving from general purpose components to an ASIC is expected to bring a reduction in power consumption, which can immediately translate into using a smaller capacity; and thus a smaller-sized battery—not only for the ULTEEM but also for the ULTEEMNite device.

The ULTEEMNite headband plays a central role in the acceptance of the device both by patients and clinicians. The current headband design can benefit from additional cushioning to improve comfort and ensure that the pressure is evenly distributed around the sensor zones to improve the contact with the skin. The latter aspect directly translates to improved signal quality and reliability in long-term recordings. Material selection for a new headband with additional cushioning will also focus on avoiding increased sweating during sleep and preserving washability of the current headband, a very important practical characteristic. While sweating initially works in favor of dry electrodes, reducing the skin-electrode impedance, excessive sweating, in addition to the comfort issue, can degrade signal quality. Sweat can create a low-impedance path through the surface of the skin, which can degrade the signal quality. The current ULTEEMNite headband was fabricated with a very thin and breathable fabric to avoid excessive sweating. Our initial studies do not show signal degradation due to sweating during the night; however, further tests under different climatic conditions are needed to form conclusions.

From a clinical point of view, it is highly important that recent studies have implied that epileptic events, in particular those occurring during sleep [29,30,31], may promote neurodegeneration [32,33,34,35]. The hypothesis states that detecting this pathological electrical brain activity early on and then initiating treatment with antiseizure medication might slow down the neurodegenerative processes and cognitive decline. Considering that in these studies the temporal lobes have been demonstrated to be particularly epileptogenic, it will be important to add more electrodes to ULTEEMNite to be able to simultaneously record not only from the frontal region, but also from both temporal brain regions.

Furthermore, not only epileptiform activity, but also sleep disturbances, per se, are established risk factors for dementia [36,37,38,39]. Thus, we expect that user-friendly, non-obtrusive devices such as ULTEEMNite that allow us to assess sleep, epileptiform activity, and their intricate relationships [40] for longer time periods will become increasingly helpful, not only to inform epilepsy care but also for the prevention, diagnosis, and monitoring of neurodegeneration. 

## Figures and Tables

**Table 1:** Most important requirements for the electronics design of the ULTEEM and ULTEEMNite systems.

Requirement	Unit	Value
Patient auxiliary current (DC)	µA	<10 NC ^1^
Patient auxiliary current (AC)	µA	<100 NC ^1^
Lower cut-off frequency of frequency response	Hz	<0.5
Higher cut-off frequency of frequency response	Hz	>50
Input-referred noise within the acquisition bandwidth	µV_p-p_ ^2^	<6
Input impedance at 10 Hz	GΩ	>0.5
Input leakage current	pA	<100

^1^ NC: normal condition as defined by [12]: condition in which all means provided for protection against HAZARDS are intact. ^2^ µV_p-p_: microvolts peak-to-peak.

**Table 2:** Summary of verification of requirements.

Requirement	Unit	Measured Value
Patient auxiliary current (DC)	µA	<0.1 ^1^
Patient auxiliary current (AC)	µA	<0.1 ^1^
Lower cut-off frequency of frequency response	Hz	<0.5
Higher cut-off frequency of frequency response	Hz	>50
Input-referred noise within the acquisition bandwidth	µV_p-p_	<5.5
Input impedance at 10 Hz	GΩ	>0.53
Input leakage current	pA	<72

^1^ Measurement limit of the instrument.

## Data Availability

The raw data that support the findings of this study are available from the corresponding author, upon reasonable request.
